# Development of antibacterial neural stimulation electrodes via hierarchical surface restructuring and atomic layer deposition

**DOI:** 10.1038/s41598-023-47256-9

**Published:** 2023-11-13

**Authors:** Henna Khosla, Wesley Seche, Daniel Ammerman, Sahar Elyahoodayan, Gregory A. Caputo, Jeffrey Hettinger, Shahram Amini, Gang Feng

**Affiliations:** 1 Department of Mechanical Engineering, Villanova University, Villanova, PA 19085 USA; 2 Pulse Technologies Inc., Research and Development, Quakertown, PA 18951 USA; 3 Department of Chemistry and Biochemistry, Rowan University, Glassboro, NJ 08028 USA; 4 Department of Biomedical Engineering, University of Southern California, Los Angeles, CA 90089 USA; 5 Department of Physics and Astronomy, Rowan University, Glassboro, NJ 08028 USA; 6 Biomedical Engineering Department, University of Connecticut, Storrs, CT 06269 USA

**Keywords:** Biomedical materials, Surface patterning

## Abstract

Miniaturization and electrochemical performance enhancement of electrodes and microelectrode arrays in emerging long-term implantable neural stimulation devices improves specificity, functionality, and performance of these devices. However, surgical site and post-implantation infections are amongst the most devastating complications after surgical procedures and implantations. Additionally, with the increased use of antibiotics, the threat of antibiotic resistance is significant and is increasingly being recognized as a global problem. Therefore, the need for alternative strategies to eliminate post-implantation infections and reduce antibiotic use has led to the development of medical devices with antibacterial properties. In this work, we report on the development of electrochemically active antibacterial platinum-iridium electrodes targeted for use in neural stimulation and sensing applications. A two-step development process was used. Electrodes were first restructured using femtosecond laser hierarchical surface restructuring. In the second step of the process, atomic layer deposition was utilized to deposit conformal antibacterial copper oxide thin films on the hierarchical surface structure of the electrodes to impart antibacterial properties to the electrodes with minimal impact on electrochemical performance of the electrodes. Morphological, compositional, and structural properties of the electrodes were studied using multiple modalities of microscopy and spectroscopy. Antibacterial properties of the electrodes were also studied, particularly, the killing effect of the hierarchically restructured antibacterial electrodes on *Escherichia coli* and *Staphylococcus aureus*—two common types of bacteria responsible for implant infections.

## Introduction

Over the past several decades, millions of patients across the world have depended on implantable neurostimulation (or neuromodulation) devices for treatment of various cardiac and neurological disorders^1–9^. These devices function by inhibition, stimulation, modification, or alteration of neural tissue through targeted delivery of an artificial electrical stimulation to specific neurological sites in the body^9^. The electrical stimulation occurs through transfer of an external electrical signal from a neurostimulator^10^ or an implantable pulse generator (IPG)^11^ to an implanted electrode or electrode array and then causing a change in response within the neural activity^11–13^.

Electrodes and electrode arrays^3,14^ are fundamental to the functionality of neurostimulation devices and thus are designed and manufactured with specific sizes, geometries, profiles, material composition, surface properties, as well as electrical, electrochemical, and mechanical properties to match the biological requirements of their intended applications^3,15–19^. Electrodes or electrode arrays for sensing applications are often required to exhibit low impedance, while high charge storage and charge injection capacity and specific capacitance are desired for neurostimulation applications and for cardiac rhythm management devices, respectively^12,14,20,21^. These specific requirements have resulted in transformations in these devices and their capabilities. In recent years, there has also particularly been a trend towards device miniaturization since smaller implantable devices are desired for compatibility with normal human activities and to enhance patient comfort^22,23^.

Enhancing the electrochemical surface area (ESA) of the electrodes not only improves the electrochemical performance of the electrodes by increasing charge storage/injection capacity and specific capacitance and reducing impedance, but also enables a decrease in geometric surface area (GSA) of the electrodes to achieve miniaturization^14,24,25^. In a recent work^12^, we demonstrated that the electrochemical performance of femtosecond-laser hierarchically-restructured Pt10Ir alloy electrodes can be tuned to yield unprecedented electrochemical performance that significantly exceeds those reported in the literature.

Surgical site-infections impact 2–4% of all patients undergoing surgery, and are the primary cause of hospital readmission post-surgery, and account for ~ 20% of all hospital acquired infections^26–28^. Notably, over half of these infections can be attributed to indwelling medical devices, with specific infection rates varying based on the type of device^29–32^. Once the infection occurs, the bacteria are often able to form biofilm structures, a 3D superstructure containing both living bacterial cells and a secreted extracellular polysaccharide layer which provides protection to the bacterial cells imbedded within^23,33,34^. These biofilms are notoriously resistant to traditional small molecule antimicrobials, a problem which is heightened by the overall increase in antimicrobial resistance^35^. The problem of antimicrobial resistance is recognized as a major threat to human health by both the CDC and the WHO^36^. Taken together, the combination of these emerging factors highlights the need to develop new and alternative strategies to combat infections, such as development of medical devices with novel antimicrobial properties^37–39^.

For safe and long-term application of implantable devices, it is necessary to endow the implant with antimicrobial properties to resist microbial-induced infections^40,41^. However, in the case of devices used in neurostimulation applications, the electrodes and electrode arrays that are at the interface of the device and the biological environment, e.g. the widely used electrode materials such as Pt10Ir alloy and coatings such as Titanium nitride (TiN) or Iridium oxide (IrO_2_)^12,42–44^ do not exhibit significant antimicrobial activity^40,41^. Bacteria and biofilm generation on long-term implantable medical devices for neurostimulation applications, e.g., implantable cardiovascular devices, can occur frequently and cause serious infections, which can lead to death^40,41^. Therefore, new methodologies for preventing and eliminating bacteria and biofilm formation are critical and needed.

Various biocompatible antimicrobial materials, e.g. copper oxide (CuO), are capable of inhibiting and/or even eliminating bacteria and biofilms^40,41,45,46^. However, these antimicrobial materials are normally not good electrical conductors and have poor mechanical properties, therefore, they cannot be directly used as electrodes in neurostimulation applications. Creating an antimicrobial surface coating on long-term implantable electrodes can be a promising approach for establishing an antimicrobial barrier, which actively prevents and eliminates the formation of bacteria and biofilms. It should also be noted that antibacterial coatings may be applied universally regardless of whether they are deposited on implantable electrodes or leads. Therefore, this study provides pivotal insights to use the same coating method to offer antibacterial properties and infection prevention for the leads by coating them with similar antibacterial coatings.

**Figure 1:**
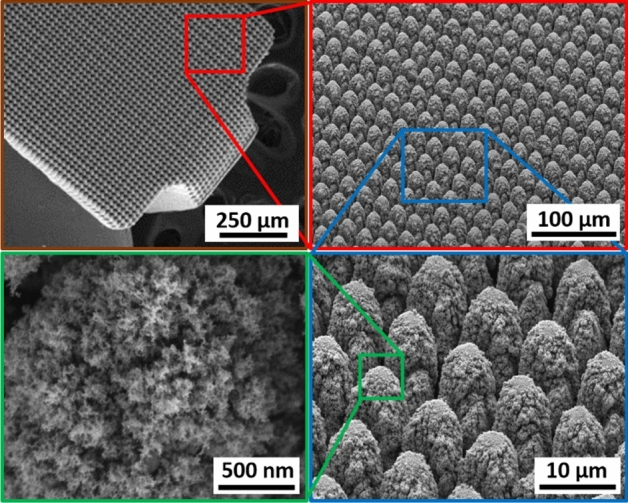
SEM micrographs of the hierarchical surface structure induced on the surface of a Pt10Ir alloy electrode used for a paddle-lead spinal cord stimulation electrode array^12^.

Hierarchically restructured Pt10Ir electrodes used in neurostimulation applications consist of topographic features spanning a variety of length scales including coarse-scale rough structures (~ 1–100 µm) and a finer nanostructure subset (~ 5–100 nm) on top of the coarse structures (Fig. 1) essential for the high electrochemical performance of these electrodes^12^. Standard coating approaches would render a subset of the increased surface area achieved by hierarchical surface restructuring inaccessible, thus reducing the desired electrochemical performance. Therefore, to minimize the impact of the antimicrobial coatings on the performance of these electrodes, the coating must be thin and conformal. Low thickness is essential for minimal impact on the hierarchical structure, and electrochemical performance such as charge storage capacity, specific capacitance and impedance. Moreover, good conformality is essential for the complete antimicrobial coverage of the complex hierarchical structure of these electrodes. Furthermore, to release the optimal amount of antimicrobial ions to the surgical site, it is important to precisely control the thickness of the antimicrobial coating: (1) the coating should be thick enough to actively prevent biofilm formation during the healing period, and (2) the coating should be thin enough to be fully dissolved after completion of healing to minimize the antimicrobial ions’ impact on the long-term health. It should be noted that this study focuses on demonstrating the feasibility of using atomic layer deposition (ALD) technique to deposit conformal and thin antibacterial coatings on complex hierarchically restructured surfaces of electrodes for neurostimulation and cardiac rhythm management devices. Investigation of the optimum ALD film thickness will be a future work.

However, achieving these two very essential attributes of the surface coating—i.e. low thickness and conformality—is extremely challenging for conventional coating techniques, such as physical vapor deposition (PVD) and chemical vapor deposition (CVD), due to their line-of-sight attributes and/or difficulty of atomic-level thickness control^47^. On the contrary, use of an atomic layer deposition (ALD) technique is ideal (and perhaps the only known suitable technique) to achieve thin and conformal coatings with excellent thickness-control and repeatability in a manufacturing environment due to the intrinsically self-limiting coating mechanism of the ALD technology^47^.

## Objectives

**Figure 2:**
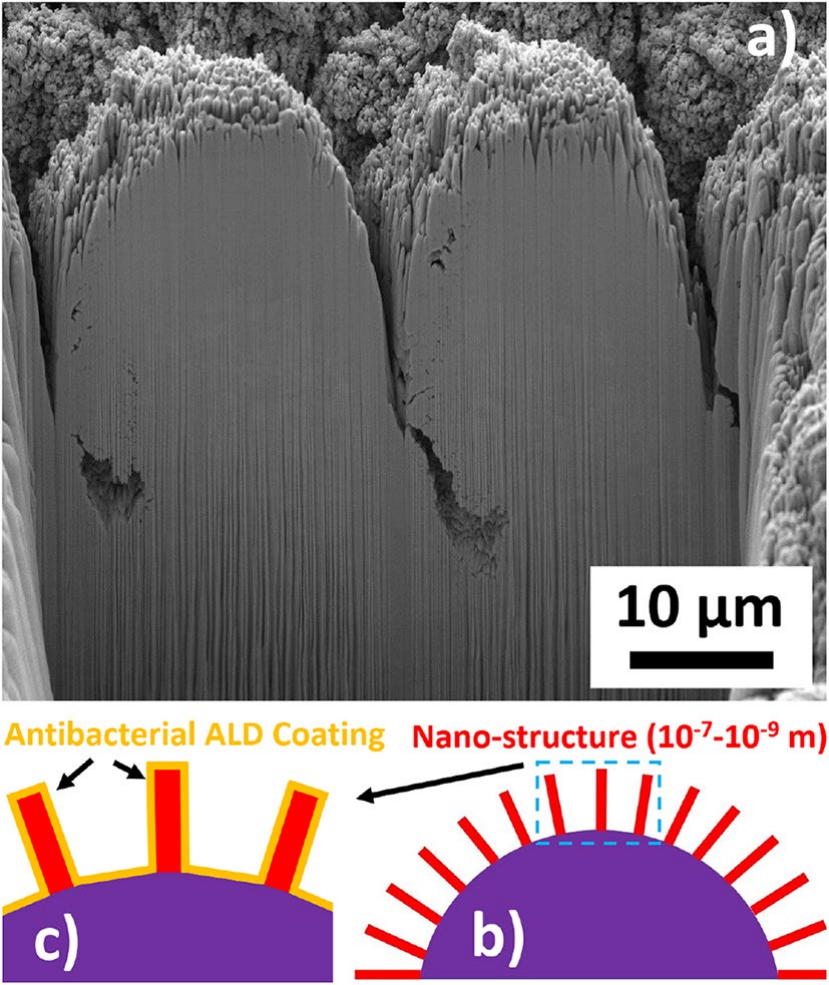
(**a**) Focused ion beam (FIB) cross section of a hierarchically restructured Pt10Ir alloy electrode^12^ showing the coarse-scale structure of peaks and valleys and the finer nano-structure subset on top of the coarse structures; (**b**) schematic of a hierarchical surface structure showing the nano-structures (red) on top of the microstructures (purple); and, (**c**) schematic of an ultra-thin ALD coating forming a conformal antibacterial layer (orange). *Note* Figure 1 for the high-resolution SEM image of the finer nano-structure subsets on top of the coarse structures of the hierarchically restructured surface of paddle-lead spinal cord stimulation electrodes.

It is hypothesized that the ALD technique can efficiently deposit conformal antibacterial thin films on the nano-structures induced on the surface of hierarchically restructured Pt10Ir electrodes, as illustrated in Fig. 2. To test this, a two-step development process was used in this work. Electrodes were first hierarchically restructured using femtosecond-laser hierarchical surface restructuring (HSR™) technology^12^. In the second step of the process, ALD was utilized to deposit thin and conformal copper oxide (Cu_x_O) films on the hierarchical surface structure of the electrodes to impart antibacterial properties to the electrodes. Subsequently, antibacterial properties and electrochemical performance of the ALD coated electrodes were evaluated. Antibacterial properties of the electrodes were studied, particularly, on the killing effect of the antibacterial electrodes on *Escherichia coli* and *Staphylococcus aureus*—two common types of bacteria responsible for infections on implanted devices. This work will demonstrate that the Cu_x_O ALD thin films deposited on hierarchically restructured Pt10Ir electrodes exhibit significant antibacterial properties while having minimal impact on the electrochemical performance of the electrodes. In addition, morphological, compositional, and structural properties of the ALD Cu_x_O coated hierarchically restructured Pt10Ir electrodes were studied using multiple modalities of microscopy and spectroscopy.

## Materials and methods

### Femtosecond laser hierarchical surface restructuring

Full details of hierarchical surface restructuring technique are outlined in a previous work^12^. In summary, a series of flat 0.3 mm thick Pt10Ir electrodes (6 and 10 mm diameter disc-shaped electrodes for electrochemical measurements and antibacterial studies, respectively, and 1 cm by 1 cm square electrodes for microscopy and spectroscopy) were hierarchically restructured and laser cut using a femtosecond laser system. The laser system used was a diode pumped Yb:YAG solid state laser (Coherent StarFemto, Santa Clara, CA) that generates 300 fs pulses with a central wavelength of 1030 nm. The experiments were performed in air, under ambient conditions. Surface patterns were created via a graphical editor (Visual Laser Marker provided by Coherent), tied into the axis controls, and the beam path was directed using a deflection head. Electrodes were mounted onto a porous ceramic vacuum plate (PhotoMachining, Inc., Pelham, NH) mounted on a tip-tilt stage (Edmunds Optics, Barrington, NJ) on an XYZ-translation stage. The electrodes were leveled to within 5 µm delta across the surface using an optical non-contact displacement transducer (Micro Epsilon, Ortenburg, Germany). Electrodes were brought directly under the deflection head to minimize the incident angle.

### ALD coating process

There are mainly two types of copper oxides: cupric oxide (CuO) and cuprous oxide (Cu_2_O)^48–50^, which will be referred to as Cu_x_O hereafter. Cu_2_O and CuO are semiconductors with significant electronic conductivity^48–50^, which is beneficial as potentially conductive antibacterial coatings for electrodes in neurostimulation applications. Cu_x_O also has significant antibacterial properties^40,41,45,46^. Copper (Cu) is an essential element to human health^51^, and also human tissue shows much larger resistance to Cu compared to micro-organisms^52–54^. Cu ions also play a crucial role in modulating wound healing process^55,56^, therefore from a biosafety and biocompatibility perspective, Cu_x_O, in small quantities^57^, is a safe choice to be applied on long-term implantable electrodes for neurostimulation and cardiac rhythm management device applications. There are various deposition techniques for thin film deposition of Cu_x_O, e.g. spin coating^58,59^, sputtering^60–62^, pulsed laser deposition^63,64^, solution processing^65,66^, and ALD^48–50^. ALD is a sequential and self-limiting chemical deposition process^47^ where the substrate is brought to various precursors and co-reactants followed by inert gas purges. Due to the ALD’s self-limiting process, the ALD films are highly conformal, uniform and well controlled even for substrates with a complex morphology^47^. The previous studies on Cu_x_O ALD deposition are limited^48–50^, and a recent study demonstrated feasibility of depositing Cu_x_O ALD films using plasma-enhanced ALD (PEALD)^50^. However, due to plasma radical recombination, PEALD is known to have difficulty to form conformal coatings on hierarchical surfaces^47,67^. We investigated here that thermal ALD using ozone as the co-reactant can efficiently create conformal Cu_x_O ALD films. In this work, Cu_x_O films were grown using a Veeco Fiji G2 system (Veeco-CNT, Waltham, MA, USA). Cu(dmap)_2_, namely, (bis(dimethylamino-2-propoxide) copper(II)), was used as the Cu-containing precursor (Ascensus-Strem, Bellevue, WA, USA). The precursor source was maintained at 125 °C using a Low Vapor Pressure Delivery (LVPD) module (Veeco-CNT, Waltham, MA, USA). Argon gas was used as the carrier gas with a constant flow rate of 30 sccm. Ozone (O_3_) was used as the co-reactant. The substrate temperature was maintained at 150 °C. Each ALD cycle consisted of a 2 s Cu precursor pulse and then a 0.075 s O_3_ pulse. Three types of substrates/electrodes were used: single crystal silicon (Si) wafers, and flat as-rolled un-restructured and smooth Pt10Ir electrodes (Pt10Ir), and flat Pt10Ir electrodes processed using hierarchical surface restructuring technology (HSR-Pt10Ir). In order to distinguish the ALD-coated and uncoated regions of the substrates/electrodes for characterization purposes, Kapton tape was used to block/mask the ALD^68^ deposition, so that only the unmasked regions could be ALD-coated.

### Spectroscopic ellipsometry and atomic force microscopy

A spectroscopic ellipsometer (M-2000, J.A. Woollam, Lincoln, NE, USA) was used to analyze the thickness and optical properties of the ALD coatings. CompleteEASE™ software was used to analyze the ellipsometry data for Si substrate with Cu_x_O coating. A Park Systems AFM (Park NX10, Santa Clara, CA, USA) with pre-mounted tip (OMCL-AC160TS, Santa Clara, CA, USA) was used in non-contact (tapping) mode (NCM) to analyze surface morphology and film thickness on the masked Si wafer. Scanning was started from the uncoated region and moved towards the coated region crossing the boundary to determine the thickness difference across the boundary.

### Scanning electron microscopy, EDS analysis, and focused ion beam milling

SEM imaging was performed using a ZEISS Crossbeam 340 (ZEISS, Oberkochen, Germany). Measurements of composition were performed using energy dispersive spectroscopy (EDS) in a Thermo Fisher Scientific Apreo S (Brno, Czech Republic). The Apreo is configured with a thermally assisted field emitter and an Oxford Instruments X-MAX 50 EDS system (High Wycombe, United Kingdom) coupled with Aztec v3.3 software capable of mapping and selected area scans. All EDS data was collected using 10 keV electrons that focused on the M_a_-lines for Pt and Ir, the L_a_-line for Cu, and the K_a_-lines for O and Si. Samples were masked with Kapton tape so that the interface between the Cu_x_O coated and uncoated regions could be clearly defined. Compositional EDS maps of Cu_x_O coated and uncoated regions were collected on Si substrates and HSR-Pt10Ir electrodes. To investigate the depth of Cu_x_O penetration into the HSR-Pt10Ir electrodes, cross-sections were milled across the coated/uncoated interface using a Tescan S8252X Plasma Focused Ion Beam (FIB) system (Brno-Kohoutovice, Czech Republic) with a Xe plasma. Xe-ions were accelerated with a 30 kV potential difference. Cross-sections were milled and imaged in the dual beam system. Subsequent measurements of composition of the materials were performed using the Apreo system with tilt stage and selected area scans. After tilting so that there was a line-of-sight path between the milled region of interest and the X-ray detector, selected area compositional scans were collected to approximately determine the depth of the Cu-signal in the restructured electrodes.

### Grazing-incidence X-ray diffraction and X-ray photoelectron spectroscopy

Grazing-incidence X-ray diffraction (GIXRD) using a Rigaku XRD system (Rigaku SmartLab X-ray diffractometer, Woodlands, TX, USA) with angle of incidence of ~ 0.5°–1° was performed. X-ray generator of 2.2 kW power was used with Cu Kα X-ray tube. Parallel Beam Optics was used out of two Bragg Brentano and Parallel Beam optics. Scanning was done from 30° to 50°. X-ray photoelectron spectroscopy (XPS) compositional analysis was performed using a PHI VersaProbe 5000 (Chanhassen, MN, USA). Monochromatic Al Kα source of 1486.2 eV was used. The XPS spot size was 200 µm and calibration was performed using C–C component of C 1 s peak at 284.8 eV. Cu 2p spectra were measured between 925 to 965 eV. Casa XPS software was used to analyze spectra and Shirley type backgrounds were used.

### Electrochemical measurements

**Figure 3:**
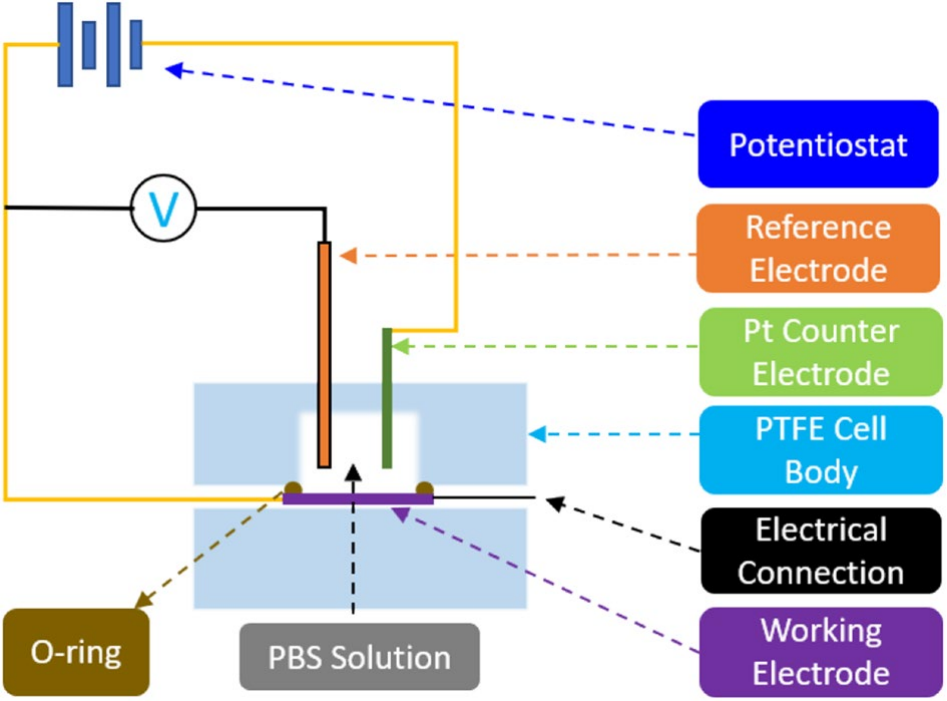
Schematic of the test setup used for CV and EIS measurements.

Cyclic voltammetry (CV) and electrochemical impedance spectroscopy (EIS) were used to measure charge storage capacity (CSC), specific capacitance and impedance^12,14,69–71^. Five different sets of electrodes were produced and used for CV and EIS measurements: Pt10Ir electrodes processed using hierarchical surface restructuring technology (HSR-Pt10Ir) tested as control and labelled as “Control”, and four sets of HSR-Pt10Ir electrodes coated with Cu_x_O ALD coatings at 5.0 nm, 9.5 nm, 12.5 nm, and 24 nm (HSR-Pt10Ir-5 nm labeled as A_1_, HSR-Pt10Ir-9.5 nm labelled as A_2_, HSR-Pt10Ir-12.5 nm labelled as A_3_, and HSR-Pt10Ir-24 nm labelled as A_4_, respectively). The voltage in the CV tests was restricted and determined by the water window representing the potential-range where oxidation or reduction currents will not lead to formation of hydrogen or oxygen at the electrode/tissue interface (− 0.6–0.8 V vs. a Ag/AgCl reference electrode)^12,14^. Both CV and EIS tests were performed in a three-electrode Teflon® plate cell (Fig. 3), comprising a Ag/AgCl reference electrode (ALS-Co Ltd., RE-1B, Tokyo, Japan), a coiled Pt counter-electrode, and the working electrodes.

The geometric surface area (GSA) of the working electrodes in the cell was 0.09 cm^2^. The electrolyte used was a commercially available phosphate-buffered saline (PBS) solution (Blood Bank Saline, Azer Scientific, Morgantown, PA). All potentials were recorded with respect to the Ag/AgCl reference electrode. All CV tests were performed at room temperature and at a 50 mV/s voltage sweep rate (ν) between potential limits of − 0.6 V and 0.8 V, beginning at open-circuit potential (OCP) and sweeping in the positive direction first. As outlined earlier, potential windows were selected to ensure water electrolysis did not occur. EIS measurements were performed at OCP and measured over a frequency range of 0.1–10^5^ Hz using a 10 mV root-mean-square (V_rms_) sinusoidal excitation voltage amplitude about a fixed potential between − 0.6 V and 0.8 V. All CV and EIS measurements were performed using a Gamry potentiostat (5000E interface, Warminster, PA) and the vendor supplied software. All data reported for CV and EIS are an average of 3 electrodes per condition, tested 3 times, i.e., a total of 9 measurements. Specific capacitance was calculated using CV/EIS data and common Randles model.

### Inductively coupled plasma mass spectrometry

Electrodes were tested for static release of copper ions into solution using inductively coupled plasma mass spectrometry (ICP-MS) as described previously^72^. Each electrode was submerged in 10 ml liquid medium and incubated at 37 °C with shaking (225 RPM) for a total period of 70 min. At 10-min intervals, 0.5 ml aliquots from these samples were removed and mixed with 5 ml 1% nitric acid, which was subsequently injected into an Agilent 7900 ICP-MS for ion release analysis. Ion concentrations were determined by comparison to a standard curve ranging from 62.5 to 4000 ppb using Aristar Copper ICP standard. All samples were tested in at least duplicates and data reported represent the average and standard deviation or range of results.

### Antibacterial measurements

All experiments were carried out at physiological temperature of 37 °C. Model bacterial strains of *E. coli* and *S. aureus* were used to differentiate activity against Gram-negative and Gram-positive strains, respectively. Two types of liquid media were used: Luria–Bertani broth (LB; Difco LB Broth, Miller) and nutrient broth (NB; Difco Nutrient Broth) bacterial growth media. All electrodes were tested in at least duplicate and data reported represent the average and standard deviation or range of results. Antibacterial analysis includes two types of characterizations: (1) Bacterial surface and contact activities: electrodes were tested for bacterial adhesion and contact-dependent antibacterial activity. Cultures of bacteria were inoculated from single colonies and allowed to grow overnight to stationary phase. An aliquot of this overnight culture was diluted 1:250 in fresh media and allowed to grow at 37 °C with shaking until the optical density at the wavelength of 600 nm (OD_600_) of the culture was in the range of 0.2–0.7, indicating mid-log growth phase. Next, an aliquot of the culture was diluted to 1 × 10^5^ CFU/ml in fresh growth medium and 10 μl of this diluted culture was deposited on a sample surface and incubated for 60 min at 37 °C^73^. Then, any remaining liquid was aspirated and the surface of the electrode was swabbed to isolate any remaining viable bacteria. The aspirated liquid was added to 3 ml of growth medium and allowed to incubate overnight at 37 °C with shaking. Next, the swab was streaked onto a petri dish containing culture medium, and then the swab was subsequently used to inoculate 3 ml of fresh culture medium. These cultures were then subjected to an 18-h incubation at 37 °C (with shaking for the liquid cultures). Bacterial growth was determined using direct visualization for the petri dish and the turbidity of the liquid cultures was measured using OD_600_; (2) Modified Kirby Bauer—zone of inhibition (ZOI) test: electrodes were tested for their ability to kill bacteria on semi-solid growth media surfaces using a modified Kirby-Bauer disk diffusion assay as described previously^74^. Briefly, electrodes were laid coated-side down onto solid nutrient agar pre-inoculated with *E. coli* (D31) or *S. aureus* (ATCC: 27660). The petri dish was allowed to incubate for 18 h at which point the diameter of the zones, if present, were measured. A zone of inhibition (ZOI) around the electrodes represents the failure of the bacteria to grow due to Cu ion release, with larger zones representing a greater degree of growth inhibition and/or bacterial killing.

## Results and discussion

### Morphology and microstructure

**Figure 4:**
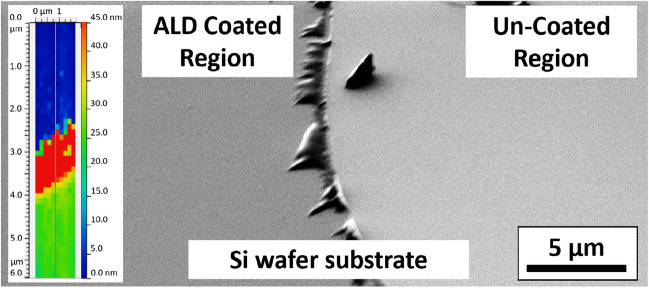
Scanning electron microscope (SEM) micrograph of a Si wafer showing the ALD coated region and uncoated region after removing the Kapton tape; inset (left) is the AFM topography contour plot showing 24.0 ± 0.9 nm film thickness measured via AFM scanning.

For morphology and microstructural characterization purposes, 950 cycles of ALD were conducted. Ellipsometry measurements of coated Si wafers measured the film to be at 21.4 nm ± 0.4 nm, indicating an average growth rate of ~ 0.022 nm/cycle. Thickness of the Cu_x_O films were also measured to be at ~ 24 nm via AFM scanning of ALD-coated Si wafer that was masked with Kapton tape after removing the Kapton tape (Fig. 4).

The AFM measured film thickness was in reasonable agreement with the ~ 21.4 ± 0.4nm thickness measured by ellipsometry. It should be noted that the coating was visible on the Si wafer with naked eye appearing in a purple color tune. The coating uniformly changes the contrast in the SEM micrographs of the Si wafer suggesting an overall uniform coating.

**Figure 5:**
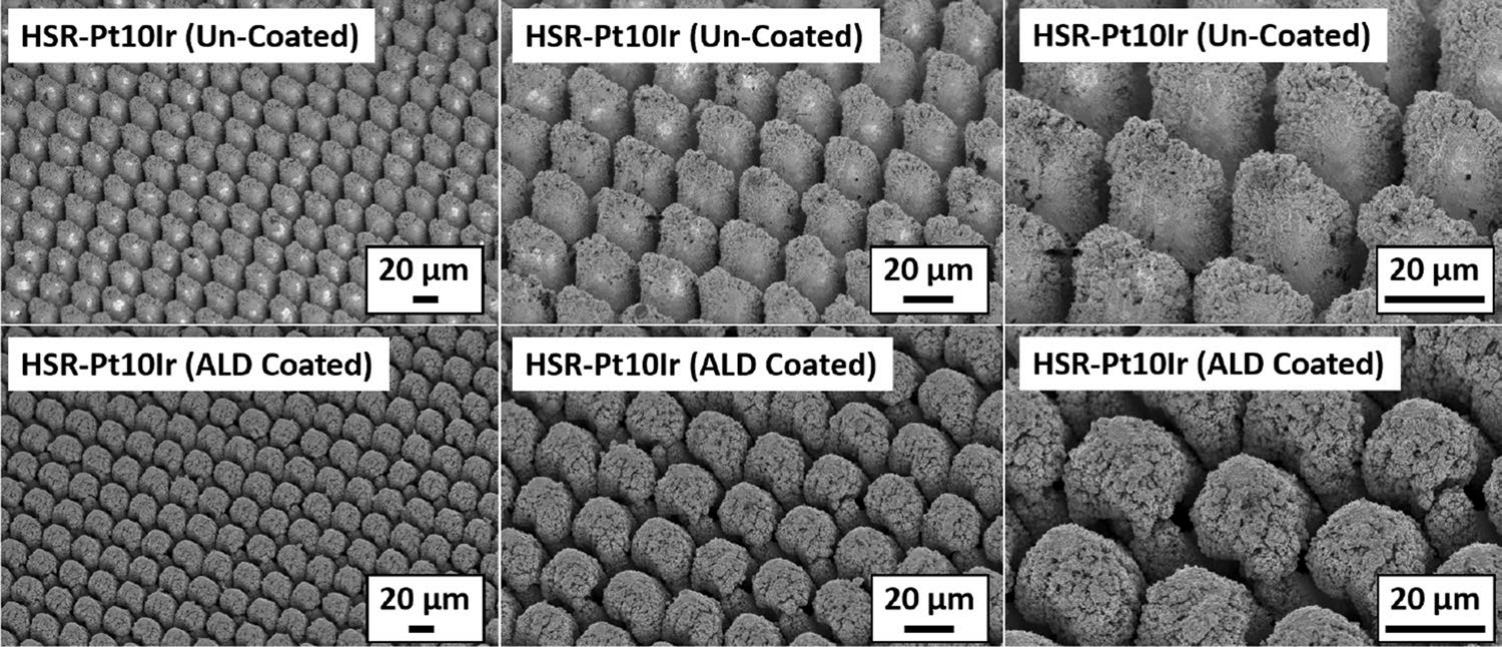
SEM micrographs of uncoated HSR-Pt10Ir electrodes (top row) and ALD coated (950 cycles at ~ 24 nm Cu_x_O film) HSR-Pt10Ir electrodes (bottom row) at various magnifications.

**Figure 6:**
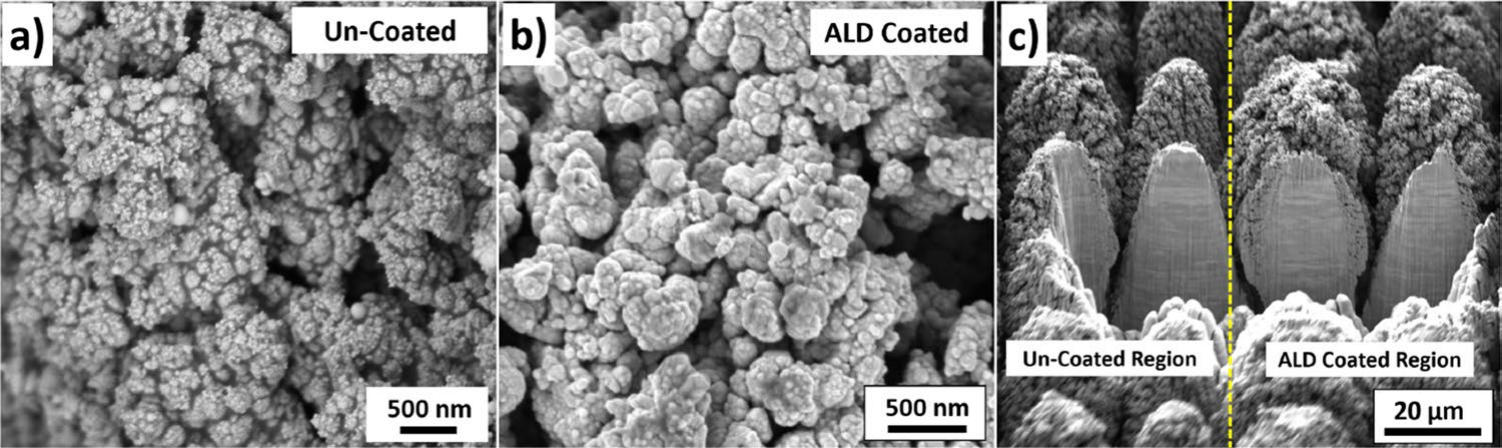
High magnification SEM micrographs of, (**a**) an uncoated HSR-Pt10Ir electrode, and, (**b**) ALD-coated HSR-Pt10Ir electrode; (**c**) Focused ion beam cross section of the masked HSR-Pt10Ir electrode showing the uncoated region (left) versus ALD-coated region (right) separated by the yellow dashed line.

Figure 5 (top row) shows the SEM micrographs of uncoated HSR-Pt10Ir electrodes at various magnifications. Figure 5 (bottom row) shows the SEM micrographs of ALD coated HSR-Pt10Ir electrodes after 950 ALD cycles at ~ 24 nm Cu_x_O film at various magnifications. Comparison of the uncoated versus ALD-coated electrodes shows that the ALD coating process has not changed the overall hierarchical surface structure of the electrodes. Figure 6 shows high magnification SEM micrographs of the nano-structured regions of the uncoated (Fig. 6a) versus ALD-coated (Fig. 6b) Pt10Ir electrodes, suggesting that the ALD-coating conformally and uniformly coats the surface, and the fine nano-structured features of the electrodes become very slightly blunted due to the ~ 24 nm ALD-coating coverage.

### Composition and structure

**Figure 7:**
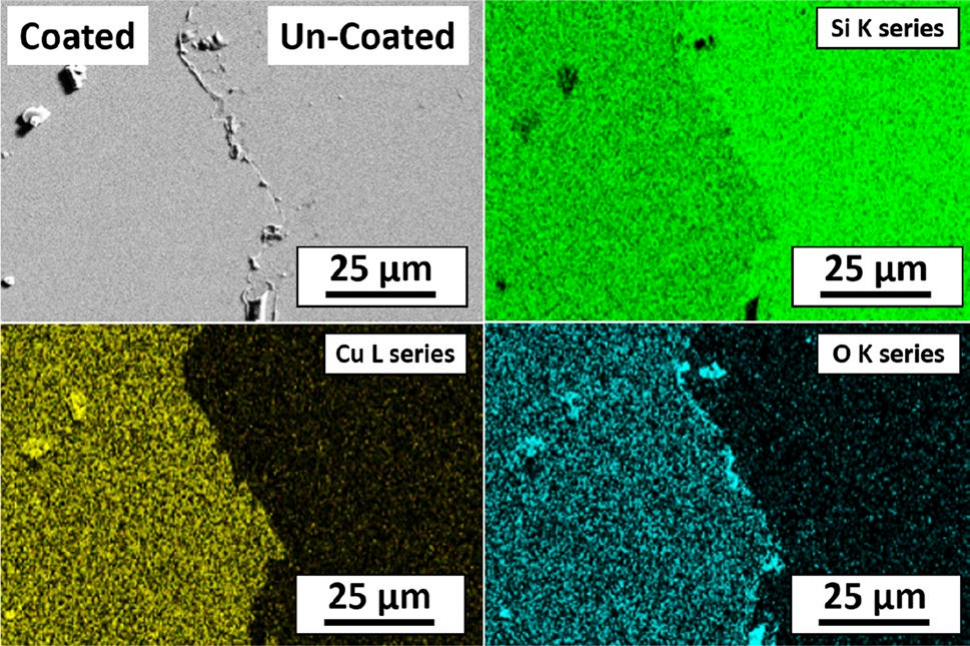
Elemental EDS maps of ALD coated Si wafer.

**Figure 8:**
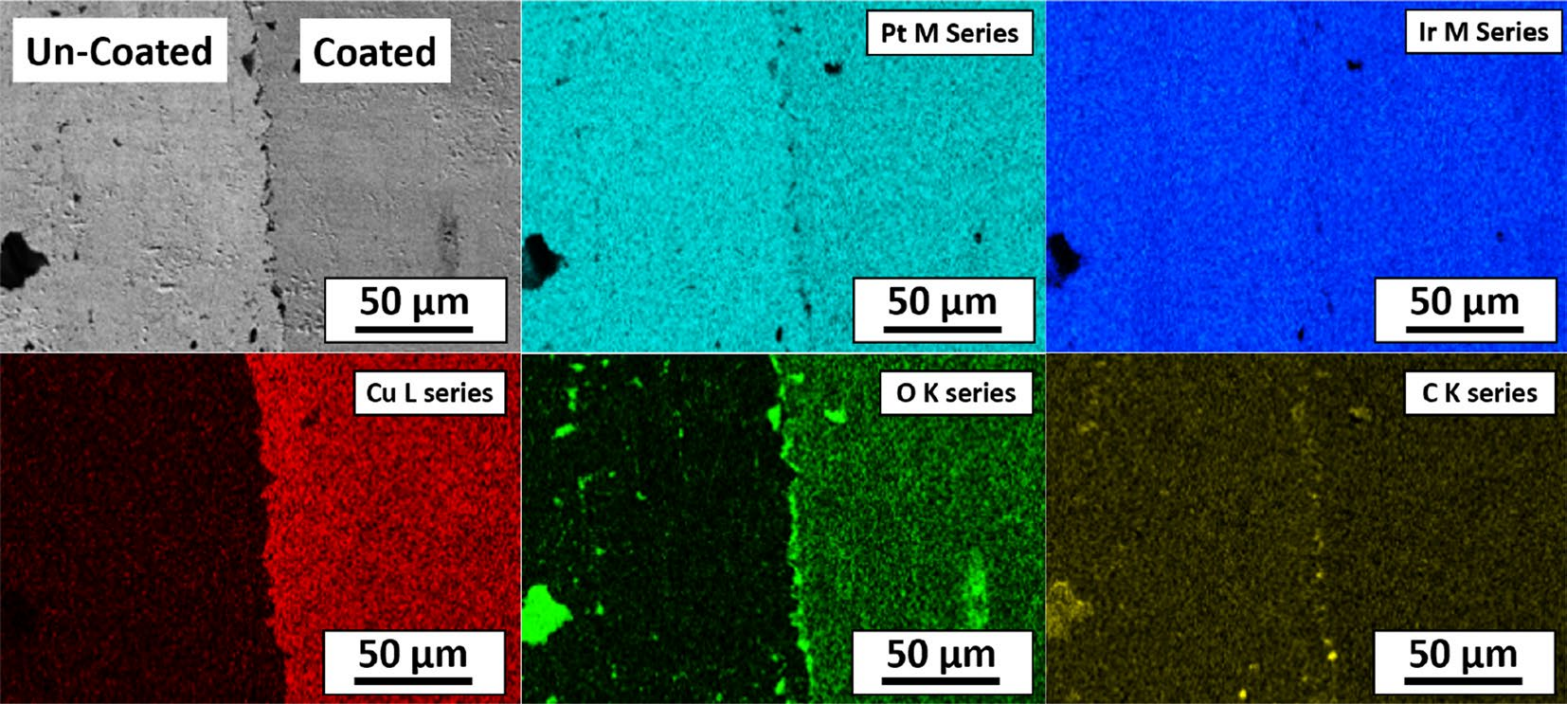
Elemental EDS maps of ALD coated Pt10Ir electrode.

**Figure 9:**
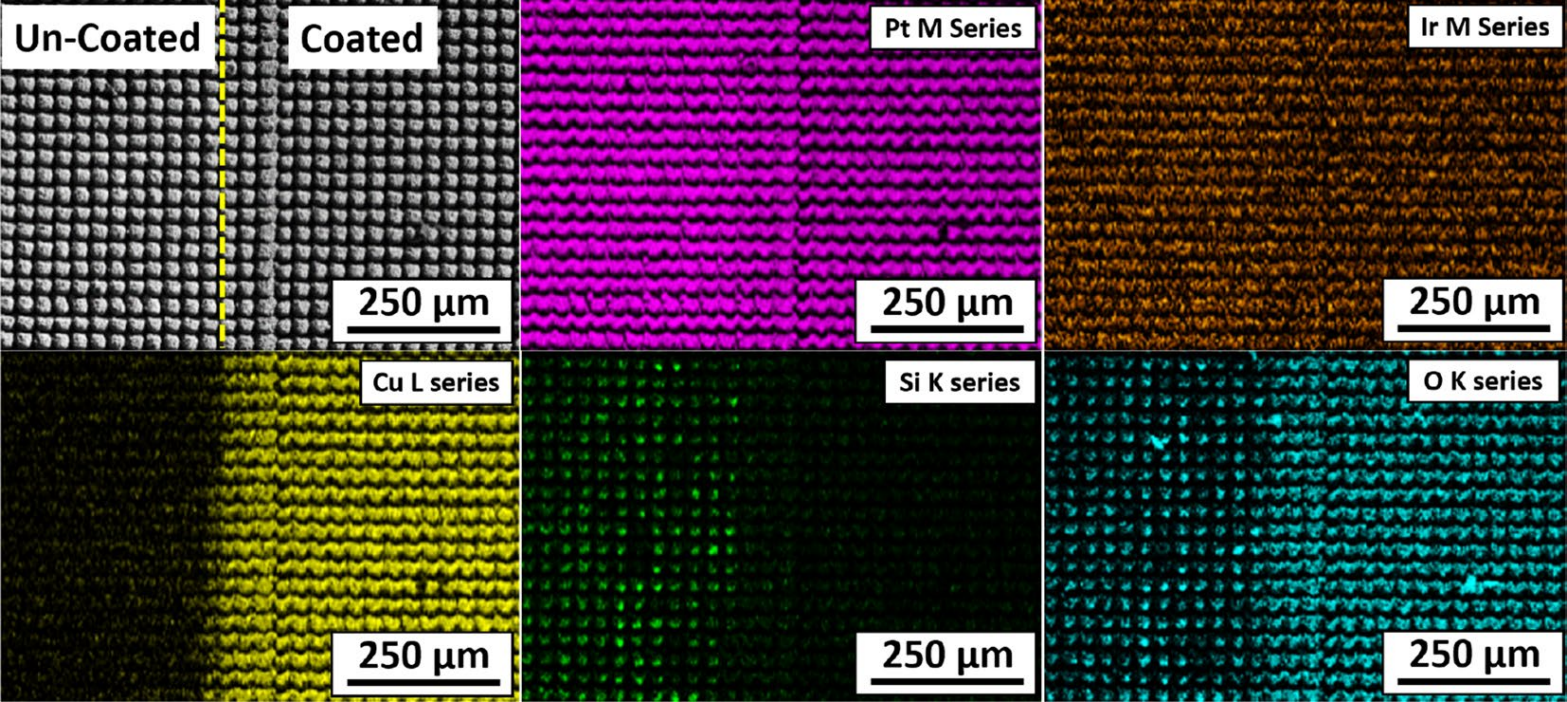
Elemental EDS maps of ALD coated HSR-Pt10Ir electrode.

Figures 7, 8, and 9 show the SEM image and corresponding elemental EDS maps of an ALD-coated Si wafer, an ALD-coated Pt10Ir electrode, and an ALD-coated HSR-Pt10Ir electrode, respectively. All three substrates were masked with Kapton tape. The EDS maps indicate that: (a) Pt and Ir universally exist as expected for Pt10Ir electrodes; (b) Cu exists only on the Cu_x_O coated regions of the electrodes; (c) Si content is lower on the Cu_x_O-coated region of the Si sample, corresponding to the Si X-rays that are generated being blocked/absorbed by the Cu-containing coating on the coated region; (d) oxygen exists on both coated and uncoated regions, where in the coated side the oxygen distribution matches well with the Cu distribution, as expected for the Cu_x_O coating, and on the uncoated side, the oxygen distribution matches well with the Si distribution as expected for the oxygen-containing silicone adhesive residues of the Kapton tape. Collectively, the EDS maps of all substrates qualitatively indicate that the ALD process can uniformly deposit Cu_x_O on the substrates and electrodes. Additionally, quantitative EDS analysis of the film on Si wafer suggests 1:1 ratio of Cu to O, suggesting that the coating is dominantly CuO, consistent with a previous study of using ozone as co-reactant^75^ as well as the XPS results discussed below.

**Figure 10:**
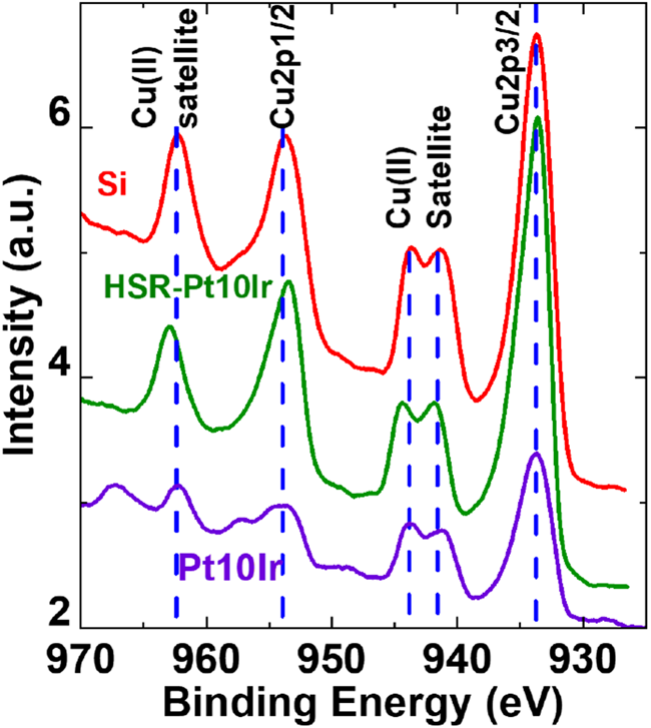
High resolution XPS spectra of Si wafer, Pt10Ir electrode and HSR-Pt10Ir electrode coated with Cu_x_O.

**Figure 11:**
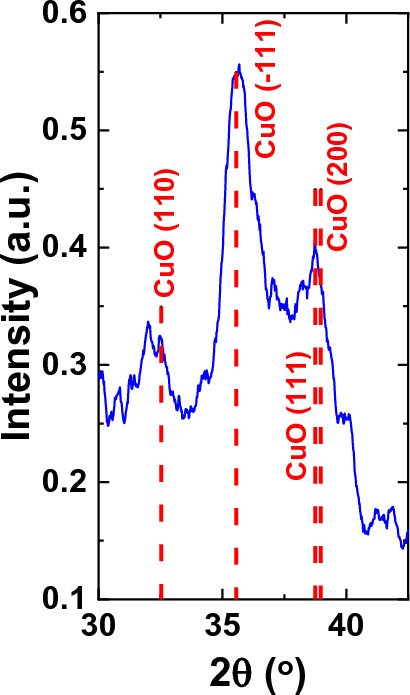
Grazing incidence X-ray diffraction pattern of Cu_x_O ALD-coated Si wafer.

Figure 10 shows the XPS Cu-2*p* spectra of Si wafer, Pt10Ir electrode and HSR-Pt10Ir electrode coated with Cu_x_O. The spectra indicates ~ 933.7 eV (Cu 2*p*_3/2_), and ~ 953.7 eV (Cu-2*p*_1/2_) peaks, and also their satellite peaks at ~ 941.5 eV and ~ 943.6 eV (Cu 2*p*_3/2_ satellite) as well as ~ 962.3 eV (Cu 2*p*_1/2_ satellite), respectively. The satellite peaks are distinguishing features of CuO^75–78^, which do not show up for metallic Cu or Cu_2_O^75–77^. Figure 11 shows the GIXRD spectra of Cu_x_O ALD coated Si wafer, again suggesting CuO^49,79^. Thus, based on EDS, XPS, and GIXRD, crystalline CuO is the dominant phase in the ALD deposited films.

### Electrochemical performance

**Figure 12:**
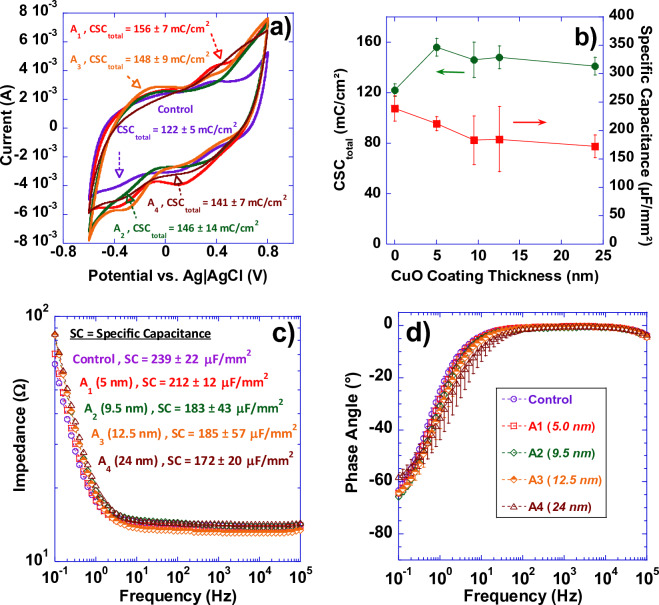
(**a**) Representative cyclic voltammograms in 1X PBS at room temperature of HSR-Pt10Ir electrode (control), and HSR-Pt10Ir electrodes coated with Cu_x_O ALD coatings at 5.0 nm (A_1_), 9.5 nm (A_2_), 12.5 nm (A_3_), and, 24 nm (A_4_) respectively; (**b**) Plots of CSC_total_ and specific capacitance (SC) of the ALD coated electrodes as a function of coating thickness; (**c**) Bode plot of impedance magnitude as a function of frequency (plotted in the 0.1–10^5^ Hz frequency range) for control, A_1_, A_2_, A_3_, and A_4_; (**d**) Nyquist plots of phase angle response of control, A_1_, A_2_, A_3_, and A_4_ electrodes plotted in the 0.1–10^5^ Hz frequency range.

Figure 12a,c display representative cyclic voltammograms and Electrochemical Impedance Spectroscopy (EIS) data for HSR-Pt10Ir electrodes (control), and HSR-Pt10Ir electrodes coated with Cu_x_O ALD coatings at 5.0 nm (A_1_), 9.5 nm (A_2_), 12.5 nm (A_3_), and 24 nm (A_4_), respectively. Figure 12b shows the plots of *CSC*_*total*_ and specific capacitance as a function of ALD coating thickness. Figure 12d shows the Nyquist plots of phase angle response of control, A_1_, A_2_, A_3_, and A_4_ electrodes plotted in the 0.1–10^5^ Hz frequency range. The EIS data (Fig. 12c) is demonstrated in bode-plot format in which the logarithm of the impedance is plotted as a function of the logarithm of frequency (plotted in the 0.1–10^5^ Hz frequency range). At high frequencies (greater than approximately 1 kHz), impedance magnitudes showed resistive behavior representing solution resistance. The CV results indicate that the total charge storage capacity (*CSC*_*total*_) of the electrodes (with uncoated “control” electrode being the baseline) slightly increases after Cu_x_O ALD deposition. This increase in *CSC*_*total*_ could likely be attributed to the semiconducting properties of CuO^48–50^. Specific capacitance of the Cu_x_O coated electrodes, however, seems to have decreased slightly compared to the uncoated control HSR-Pt10Ir electrode. The reason(s) for this slight decrease in capacitance is not yet entirely understood but it could be attributed to the slight decrease in the double layer capacitance elements in the hierarchical surface structure of the electrodes’ surface (the peaks and valleys) being slightly blunted due to increase in thickness of the Cu_x_O ALD-coating coverage from ~ 5 nm (A_1_) to ~ 24 nm (A_4_), as shown in the SEM and FIB micrographs of Figs. 5 and 6. The EIS bode plots suggest that the overall impedance behavior of the electrodes as a function of frequency seem to have remained unchanged (within experimental scatter). The response of phase angle, which is the arctangent of the ratio of the imaginary and real parts of the measured impedance, is shown in the Nyquist plots of phase angle response plotted as a function of frequency (log) (Fig. 12d). At frequencies above 100 Hz, the phase angles for all the electrodes were close to zero, which indicates the purely resistive nature of the system. At frequencies below 100 Hz, the electrodes had none-zero phase angle values decreasing to ~ − 60° as DC frequency was approached indicating the presence of reactance. Overall, the CV and EIS measurements suggest that the Cu_x_O ALD coatings have insignificant impact on the overall electrochemical performance of the electrodes post-ALD.

### Antibacterial properties

**Figure 13:**
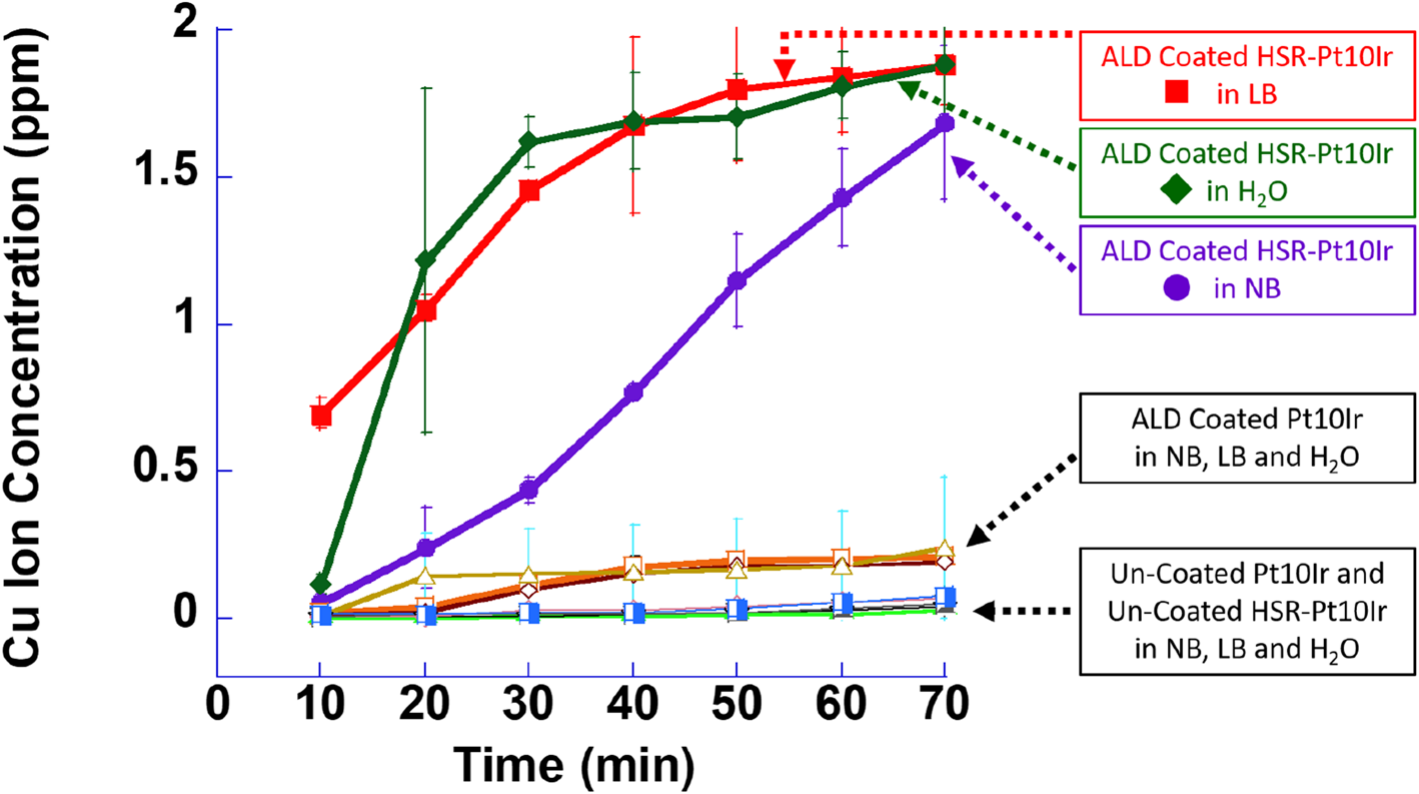
Cu ion concentration measured by ICP-MS as a function of time for Pt10Ir and HSR-Pt10Ir electrodes (ALD-Coated and uncoated) in LB, NB, and H_2_O.

CuO is known to act as an antibacterial^80,81^. The mechanism for CuO’s antibacterial capability is complex and multifold but is directly linked to the interaction of the Cu ions with the bacterium. Some possible mechanisms are: (1) Cu ions can change the charge of the surrounding medium to break and rupture cell wall membrane^82^; (2) Cu ions can alter DNA molecules and disrupt the biochemical process in bacteria^83^. When the concentration of Cu ion reaches a threshold in the culture medium, the bacteria would be killed with sufficient time^83–85^. Thus, to check the Cu release of the ALD-coated electrodes, ICP-MS measurements were conducted on Pt10Ir and HSR-Pt10Ir electrodes (ALD-coated and uncoated). Figure 13 shows Cu ion concentration as a function of time in LB, NB, and H_2_O. As expected, the uncoated Pt10Ir and HSR-Pt10Ir electrodes show no evidence of released Cu ions over time due to the absence of any CuO coating on those electrodes. It should be noted that the charge state of the Cu ions is not determined in this work, and they may exist as Cu^1+^, Cu^2+^, or a mixture of these.

Figure 13 indicates that Cu ion concentration in ALD-coated Pt10Ir electrodes for all three types of media continues to slowly increase with increasing electrode immersion time up to ~ 40 min after which the Cu ion concentration reaches a plateau of ~ 0.2 ppm. With water and LB as the media, the Cu ion concentration of ALD coated HSR-Pt10Ir electrodes rapidly increases as a function of time and reaches ~ 1.9 ppm after 70 min. In NB media, the Cu ion concentration of ALD coated HSR-Pt10Ir electrodes increase but at a slower rate and reach ~ 1.7 ppm after 70 min. This is strong evidence that hierarchical surface restructuring of the electrodes prior to ALD coating can increase the Cu ion release rate by nearly one order of magnitude due to the increase in surface area of the electrodes after restructuring^12^. The ion release experiments also clearly suggest that the CuO coating can release Cu ions into the surrounding medium.

**Figure 14:**
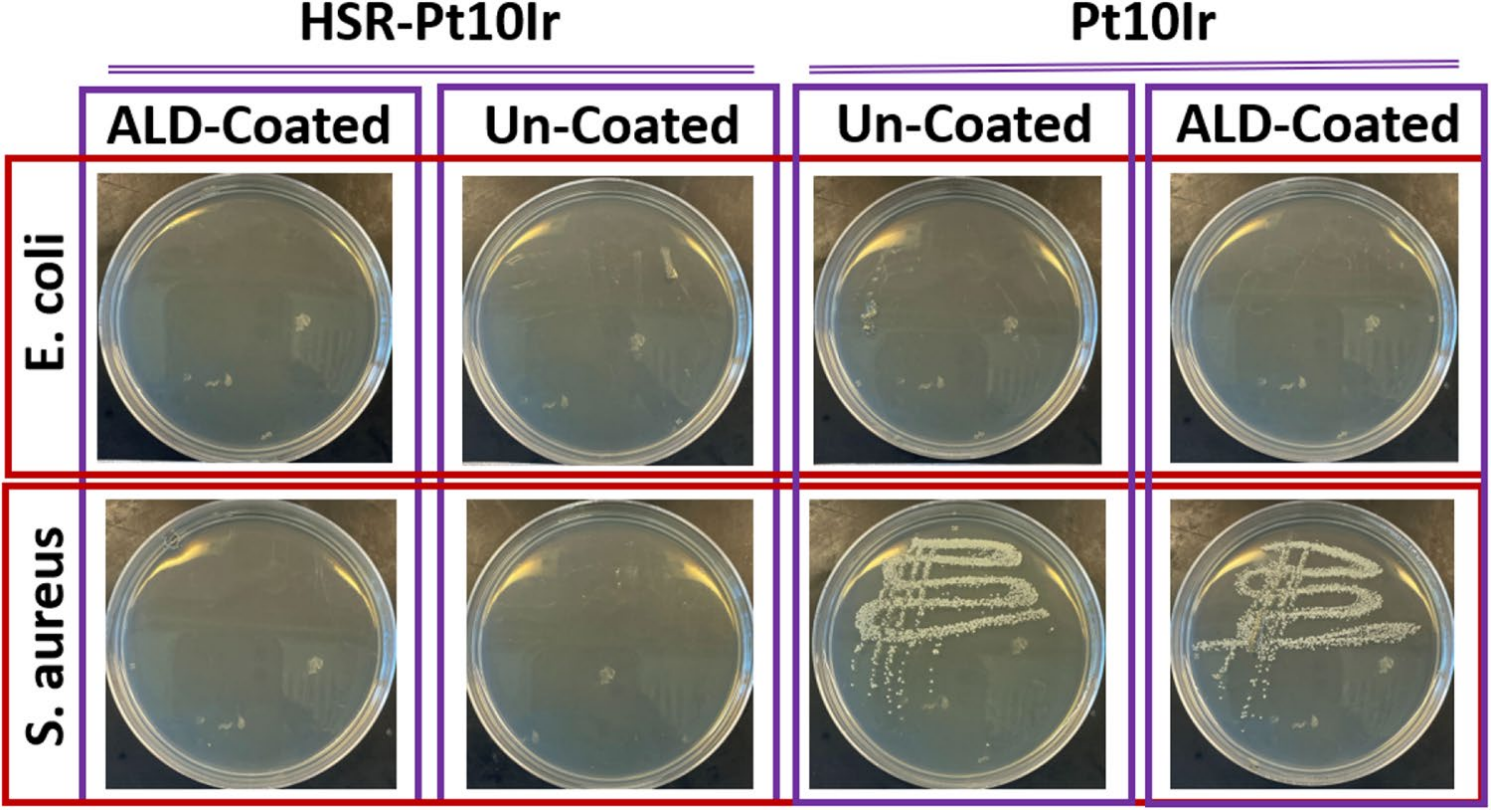
Bacterial surface and contact activities of each liquid culture (in LB) which contacted with the corresponding electrode: Agar plate analysis for the swabbing.

**Figure 15:**
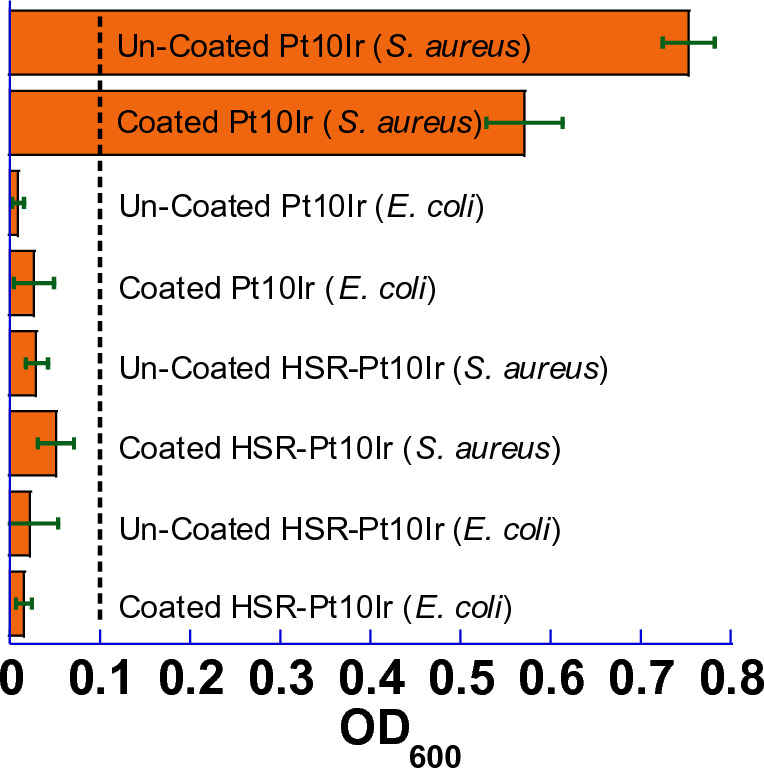
Bacterial surface and contact activities of each liquid culture (in LB): OD_600_ vs. the corresponding liquid culture, where the dash-dot line indicates the threshold (0.1) for significant bacterial development.

Bacterial growth on a swabbed petri dish and the turbidity (OD_600_) of the liquid culture inoculated after surface sampling (in LB media) are shown in Figs. 14 and 15. The results interestingly indicate that uncoated Pt10Ir electrodes show no inhibition of *S. aureus* growth but do exhibit inhibition of *E. coli* growth, suggesting that Pt10Ir itself is intrinsically antibacterial^86^ against *E. coli* but not against *S. aureus* in direct contact. On the other hand, the coated Pt10Ir electrodes showed similar behavior as their uncoated counterparts, which may be attributed to the fact that the coated Pt10Ir electrodes did not rapidly release sufficient Cu ions (see Fig. 13) to reach the required threshold concentration to inhibit the growth of *S. aureus*. This could be attributed to a reduced accessible surface area in comparison to the conformal coatings on the restructured electrodes.

Figure 14 also shows that the uncoated HSR-Pt10Ir electrode inhibited the growth of both *E. coli* and *S. aureus*, which might be the combination of antibacterial Pt10Ir^86^ surface and the nanostructured morphology resulting from hierarchical surface restructuring^87,88^. Sharp surface nanomorphology has been previously shown to be capable of killing Gram-negative (e.g., *E. coli*) and Gram-positive (e.g., *S. aureus*) bacteria in contact, possibly through physically rupturing the bacterial cell wall^87,88^. Thus, hierarchical surface restructuring of electrodes creates a hierarchical surface structure that induces bacterial-killing characteristics in contact while demonstrating high electrochemical performance.

Figure 15 shows the OD_600_ for the surface-contacting liquid culture, again indicating that only the uncoated and CuO-coated Pt10Ir electrodes did not inhibit *S. aureus* growth on contact and all HSR-Pt10Ir electrodes are antibacterial in direct contact against both *E. coli* and *S. aureus*. This is consistent with the results of bacterial growth on swabbed petri dishes (Fig. 14). Thus, only based on the study of bacterial surface and contact activities as in Fig. 14, the effect of hierarchical surface restructuring combined with CuO coating cannot be differentiated.

**Figure 16:**
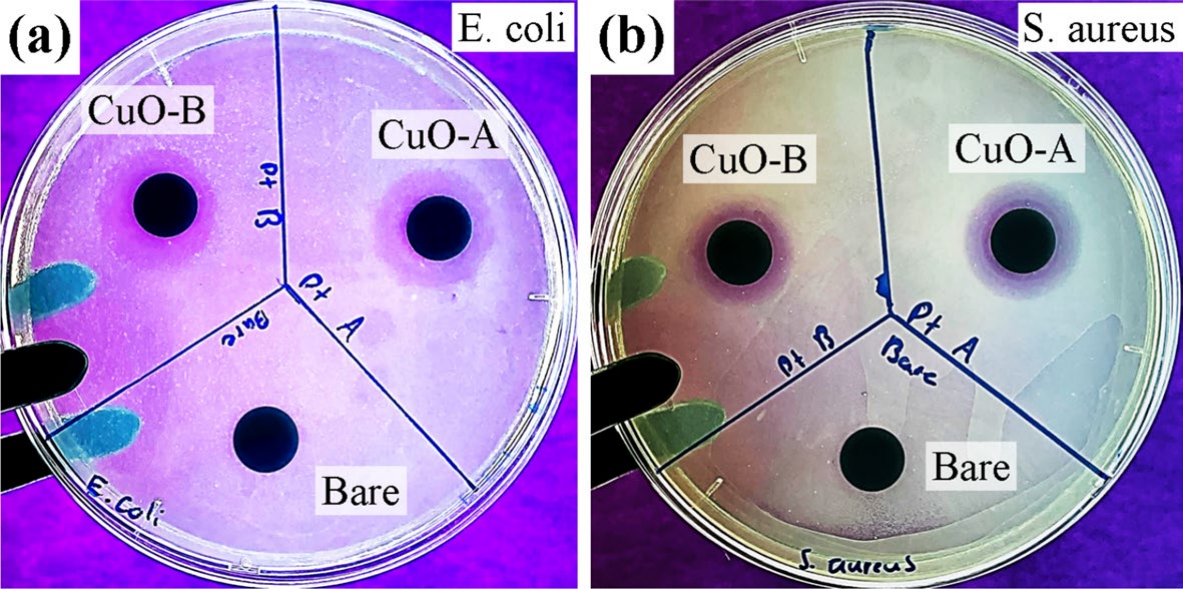
ZOI assays for HSR-Pt10Ir electrodes in NB. Two CuO coated electrodes (A and B) and an un-coated HSR-Pt10Ir electrode in (**a**) *E. coli* and (**b**) *S. aureus* were tested.

Considering the previous results, a modified Kirby Bauer method to test zone of inhibition (ZOI) was used to test the effect of CuO coatings deposited on hierarchically restructured electrodes. ZOI assays in NB are shown in Fig. 16, indicating zero ZOI for uncoated HSR-Pt10Ir electrodes and significant ZOI for coated HSR-Pt10Ir electrodes: 19.3 ± 0.2 mm for *E. coli* and 16 ± 1.5 mm for *S. aureus*. This implies that the CuO coating, i.e., the Cu ion release, is solely responsible for the formation of ZOIs. Thus, while hierarchically restructured electrodes can kill *E. coli* and *S. aureus* in direct contact mainly due to the sharp nanomorphology (Figs. 14, 15), the CuO coating on hierarchically restructured electrodes is capable of releasing sufficient antibacterial Cu ions to kill *E. coli* and *S. aureus* in the surrounding medium that is not directly contacting with the electrode surface (Fig. 16).

## Concluding remarks

In this report, we introduced a two-step process using femtosecond laser hierarchical surface restructuring (HSR™) and atomic layer deposition (ALD) for development of electrochemically active antibacterial platinum-iridium (Pt10Ir) electrodes targeted for use in cardiac rhythm management device, and neurostimulation and sensing/recording applications. We showed that after hierarchical surface restructuring, the surface of Pt10Ir electrodes undergo topographical transformations that result in electrodes with superior electrochemical performance. These topographic features span a variety of length scales including coarse-scale rough microstructures and a finer nanostructure subset on top of the coarse structures that impart high electrochemical performance to the electrodes. Through multiple modalities of microscopy and spectroscopy for surface and subsurface characterization of the ALD-coated hierarchically restructured electrodes, we demonstrated that the electrodes were coated with ultra-thin (~ 24 nm) and conformal CuO films without changing the overall hierarchical surface structure of the electrodes. The results of electrochemical measurements on ALD-coated hierarchically restructured electrodes indicated that the total charge storage capacity of the electrodes slightly and favorably increases after CuO ALD deposition, possibly attributed to the semiconducting properties of CuO. The antibacterial properties of the ALD-coated hierarchically restructured electrodes were also studied, particularly, the killing effect of the electrodes on *E. coli* and *S. aureus*—two common types of bacteria responsible for implant infections. It was shown that uncoated hierarchically restructured electrodes can kill *E. coli* and *S. aureus* in direct contact likely due to the sharp nanomorphology of the hierarchical structures on the surface of the electrodes. More importantly, it was demonstrated that the ALD-deposited CuO coating on hierarchically restructured electrodes is capable of releasing sufficient antibacterial Cu ions to kill *E. coli* and *S. aureus* in the surrounding medium that is not directly contacting with the electrode surface. Thus, this study shows that antibacterial ultra-thin and conformal CuO films can be deposited via ALD on hierarchically restructured Pt10Ir electrodes with minimal impact on their electrochemical properties, rendering the electrodes antibacterial. Since the threat of antibiotic resistance is significant and is increasingly being recognized as a global problem, the findings reported in this study are insightful and paramount for design and manufacturing of neurostimulation and cardiac rhythm management electrodes with antibacterial properties to eliminate post-implantation infections and reduce antibiotic use.

## Data Availability

All datasets used and/or analysed during the current study are available from the corresponding author on reasonable request.
